# Simulation of Wheat Productivity Using a Model Integrated With Proximal and Remotely Controlled Aerial Sensing Information

**DOI:** 10.3389/fpls.2021.649660

**Published:** 2021-03-24

**Authors:** Taehwan Shin, Jonghan Ko, Seungtaek Jeong, Ashifur Rahman Shawon, Kyung Do Lee, Sang In Shim

**Affiliations:** ^1^Department of Applied Plant Science, Chonnam National University, Gwangju, South Korea; ^2^Department of Agricultural Environment, National Institute of Agricultural Science, Wanju, South Korea; ^3^Department of Agricultural Science, Gyeongsang National University, Jinju, South Korea

**Keywords:** aerial images, crop model, remotely controlled aerial system, proximal sensing, simulation, wheat

## Abstract

A crop model incorporating proximal sensing images from a remote-controlled aerial system (RAS) can serve as an enhanced alternative for monitoring field-based geospatial crop productivity. This study aimed to investigate wheat productivity for different cultivars and various nitrogen application regimes and determine the best management practice scenario. We simulated spatiotemporal wheat growth and yield by integrating RAS-based sensing images with a crop-modeling system to achieve the study objective. We conducted field experiments and proximal sensing campaigns to acquire the ground truth data and RAS images of wheat growth conditions and yields. These experiments were performed at Gyeongsang National University (GNU), Jinju, South Gyeongsang province, Republic of Korea (ROK), in 2018 and 2019 and at Chonnam National University (CNU), Gwangju, ROK, in 2018. During the calibration at GNU in 2018, the wheat yields simulated by the modeling system were in agreement with the corresponding measured yields without significant differences (*p* = 0.27–0.91), according to two-sample *t*-tests. Furthermore, the yields simulated via this approach were in agreement with the measured yields at CNU in 2018 and at GNU in 2019 without significant differences (*p* = 0.28–0.86), as evidenced by two-sample *t*-tests; this proved the validity of the proposed modeling system. This system, when integrated with remotely sensed images, could also accurately reproduce the geospatial variations in wheat yield and growth variables. Given the results of this study, we believe that the proposed crop-modeling approach is applicable for the practical monitoring of wheat growth and productivity at the field level.

## Introduction

Wheat (*Triticum*) is a global staple food crop, a cereal grain, and a grass cultivated worldwide with broad adaptability from temperate to cold environments (Martin et al., 2005; Shewry, 2009). The effective increase in wheat productivity is of interest to farmers, researchers, shareholders, and policymakers involved in the agricultural production business (Aarts et al., 2014). Early estimations of wheat productivity and timely information regarding seasonal growth conditions are expected

to increase its productivity through cohesive crop management practices such as irrigation and nitrogen application regimes. Crop modeling and remote sensing (RS) are both conventional methodologies that, when used in conjunction, offer all the benefits of the techniques available for practical assessments of crop productivity and growth conditions (Nguyen et al., 2019). A crop model allows for sequential simulation, while RS enables consistency in the monitoring of geographic and spatial variations in crop conditions and productivity (Ko et al., 2015; Jeong et al., 2018b).

Mathematical crop growth models include a set of formulas that describe crop growth and development over a continuous scale before their maturity or harvest (Thornley and Johnson, 1990). Empirical crop models commonly comprise few equations for the requirements of simulating growth and development for a discrete period or crop yield. In contrast, mathematical crop models are formulated using additional equations to simulate seasonal crop growth based on a mathematical approach. Therefore, mathematical crop models are advantageous for simulating the seasonal patterns of crop growth and productivity and for forecasting their yields. A mathematical crop model contains growth parameters specific to cultivars and environments, and it is highly dependent on the growth and development of the canopy (Ahuja et al., 2000; Jones et al., 2003). Appropriate parameters help streamline the model for estimating variables pertaining to crop growth (Maas, 1993b). Steady simulations using a crop model require many inputs, encompassing parameters, and variables related to the environment, soil, and weather. It is also challenging to obtain adequate parameters. A mathematical crop model is often weak in terms of geographical or regional simulations of crop growth and productivity with realistic precision. This drawback is attributable to the lack of appropriate spatial and temporal information regarding canopy growth (Doraiswamy et al., 2003) and the flaws in the input data (Moulin et al., 1998).

RS is another approach that is appropriate for exploring the conditions of crop growth and development with respect to geographic and spatial variability during growing seasons (Campbell and Wynne, 2011). It is advantageous for obtaining detailed information regarding crop growth conditions from an available RS scene of the site of interest. However, when using most RS platforms, either remote-controlled aerial vehicles or satellites, it is unfeasible to deliver the necessary information with a continuous source. This is because of the restricted revisit time to the fields of interest or unfavorable environmental conditions (Moulin et al., 1998; Jeong et al., 2020). Crop growth conditions and yields can also be assessed by using the empirical relationship between a crop growth variable and the RS data obtained from various RS platforms (Bouman, 1992; Zarco-Tejada et al., 2005; Dorigo et al., 2007). Some research efforts have been devoted toward predicting and assessing crop yields based on the empirical relationship between the yield and optical RS information (Clevers, 1997; Labus et al., 2002; Kern et al., 2018). These types of empirical crop models are appropriate for evaluating growth conditions and productivity in a particular region of interest for practical reasons. However, this approach has a disadvantage in that the growth and development processes or their influences on productivity cannot be explained (Delécolle et al., 1992; Becker-Reshef et al., 2010). This approach is also likely to neglect the interaction between radiation and vegetation canopies (Bouman, 1992).

A crop model integrated with RS information can reinforce the advantages of RS and crop modeling, thereby circumventing the weaknesses of these approaches (Delécolle et al., 1992; Maas, 1992; Ko et al., 2015). This methodology can enable geospatial crop productivity monitoring and yield forecasting at different scales of croplands (Jeong et al., 2018b; Nguyen et al., 2019). Previous studies have attempted to utilize this strategy of combining crop modeling and RS (Moulin et al., 1998; Cheng et al., 2016; Huang et al., 2018, 2019; Jin et al., 2018; Nguyen et al., 2019). GRAMI (Maas, 1992), a gramineous crop model, was developed through such efforts; this model can use RS data. GRAMI (Maas, 1993a,b) is a mathematical model that requires simple crop parameters, weather variables, and RS data from any platform to simulate grain crop growth and estimate yield. The GRAMI model was further extended to simulate cotton (Ko et al., 2005, 2006), soybean (Shawon et al., 2020a), and paddy rice (Ko et al., 2015). The present version of GRAMI has been renamed as a RS-integrated crop model (RSCM), indicating that the model is updated with the abovementioned information for future simulations of various crops. The RSCM allows the monitoring of croplands at different scales, ranging from farm fields to various geographical regions (Jeong et al., 2018a,b, 2020; Yeom et al., 2018; Shawon et al., 2020a).

Recently, there has been growing interest in employing a remote-controlled aerial system (RAS) for different agricultural and industrial activities, such as field-based crop assessments and management practices (Mesas-Carrascosa et al., 2015; Jeong et al., 2016; Cai et al., 2019). The RSCM is also capable of evaluating geospatial crop growth conditions and productivity using RAS-based RS images (Jeong et al., 2018a). This study aimed to achieve an advanced management option for stable and improved wheat production with different cultivars and various nitrogen application regimes employing the modeling system. These cultivation practices were designed to explore the best management practice option based on the modeling system. We extended the RSCM to simulate geospatial wheat productivity at the field scale by using RAS-based RS data to perform the study goal.

## Materials and Methods

### Field Experiment

We carried out field experiments at two study sites—one in Jinju and another in Gwangju, South Korea—to assess the RSCM in terms of its capability of simulating wheat growth and yield during the growing seasons of 2018 and 2019. The field experiment in Jinju was performed at Gyeongsang National University (GNU; 35°8′ N, 128°5′ E; 33 m), during the wheat seasons in 2018 and 2019 to estimate model parameters and obtain datasets for evaluating the modeling scheme. The experiment in Gwangju was performed at Chonnam National University (CNU; 35°10′ N, 126°53′ E; 33 m), during the wheat season in 2018 to validate the modeling scheme. Both these study locations experience a typical East Asian monsoon climate. According to Korea Meteorological Administration (https://www.kma.go.kr/eng/), mean annual temperature and average yearly precipitation have been recorded at 13.1°C and 1,513 mm in Jinju and 13.8°C and 1,391 mm in Gwangju, respectively, over the past 30 years. It typically rains (~60%) during the summer and monsoon seasons (i.e., July–August). At GNU, the topsoil layer (0–20 cm) is categorized as sandy loam (71.4% sand, 18.8% silt, and 9.7% clay), with a pH of 5.9, organic carbon content (OCC) of 8.6 g C kg^−1^, available phosphorus (P) of 185 mg P_2_O_5_ kg^−1^, cations exchange capacity (CEC) of 6.3 cmolc kg^−1^, and total nitrogen (TN) before fertilization of 0.053 g N kg^−1^, according to National Institute of Agricultural Sciences (www.naas.go.kr/english/). At CNU, the topsoil layer (0–30 cm) is categorized as loam (43.1% sand, 30.9% silt, and 26.0% clay), with a pH of 6.5, OCC of 12.3 g C kg^−1^, P of 131 mg P_2_O_5_ kg^−1^, CEC of 14.4 cmolc kg^−1^, and TN of 1.0 g N kg^−1^.

Two wheat cultivars—Chokyung and Keumkang—were sown on October 30, 2017, and February 9, 2018, and harvested on June 25, 2018, over an area of ~714.0 m^2^ at GNU, while only Chokyung with different N treatments was seeded on October 30, 2018 and February 18, 2019 and harvested on June 10, 2019. At CNU, Chokyung was sown on February 26, 2018 and harvested on June 20, 2018, over an area of ~634.0 m^2^. Additional information regarding the cultivars can be referenced from the website of the National Institute of Crop Science (www.nics.go.kr/english/). There were two different experimental arrangements for the N treatments (recognized as specific and gradient levels) at GNU in 2019. The N treatments involved varying applications of 40 kg ha^−1^ at planting, 30 kg ha^−1^ at rejuvenation, and 0 kg ha^−1^ at initial reproduction (N40-30-0), N40-30-30, and N40-30-60. The gradient N treatment was arranged with N40-0-0, N40-10-10, N40-20-20, N40-30-30, N40-40-40, N40-50-50, and N40-60-60. All the experimental blocks at both GNU and CNU were placed in a randomized complete block design at three replications for each corresponding year. Wheat grains were sown in a row spacing of 0.2 m and a hill-to-hill spacing of 0.1 m using a mechanical seed drilling device. The N fertilizer for the standard treatment in this study was applied at 100 kg ha^−1^, spreading 40% under the soil surface as a basal dosage before seeding and being treated as a side dressing at 30% for the tillering and panicle initiation stages. Two fertilizers (P) and potassium (K), were applied at 70 and 35 kg ha^−1^, respectively. Full doses of P and K were spread under the soil surface as a basal application before seeding.

The above-ground dry mass (AGDM) and leaf area index (LAI) were considered as growth variables and measured during the main wheat development stages. LAI was measured using LAI-2200C (LI-COR Inc., Lincoln, NE, USA), which can quantify the LAI of the canopy under diffuse sunlight, even under daylight, through its light-diffusing cap and light scattering correction method, thereby affording precise and accurate results. LAI was measured on the 87, 101, 115, 130, and 144 day of year (DOY) for the autumn-seeded wheat and the 101, 115, 130, 144, and 158 DOY for the spring-seeded wheat at GNU in 2018. In 2019, LAI measurements were conducted on the 68, 81, 102, 111, and 145 DOY for the autumn-seeded wheat and the 102, 111, and 145 DOY for the spring-seeded wheat. LAI measurements at CNU were conducted on the 100, 106, 111, 117, 124, 128, 134, 141, and 149 DOY in 2018. LAI was measured three times in repetition at the same plot. Meanwhile, the plant samples were harvested for AGDM estimation. Plant samples were harvested, except for the root, at each plot on the 100, 111, 120, and 128 DOY in 2018 at CNU, Gwangju. Likewise, plant samples at GNU, Jinju, in 2018 were collected on the 72, 87, 102, 116, 129, and 144 DOY for the autumn-sown wheat and the 87, 102, 116, 129, and 144 DOY for the spring-sown wheat. Plant sampling in 2019 was conducted on the 67, 80, 102, and 109 DOY for the autumn-sown wheat and the 96, 123, and 130 DOY for the spring-sown wheat. The plant samples were then separated into their leaves, stem, and spike, and the samples were oven-dried at 70°C for 1 week, depending on the sample condition. Plant sampling was performed to estimate biomass partitioning through photosynthesis by utilizing photosynthetically active radiation.

Weather data at the study sites were recorded using automated weather stations, MetPRO (Campbell, Logan, UT, USA) at GNU and WS-GP1 (Delta-T Devices, Cambridge, UK) at CNU. The daily average mean temperature, solar radiation, and precipitation at GNU were 8.79 °C, 11.81 MJ m^−2^ d^−1^, and 2.96 mm d^−1^ during the 2018 season (October 30, 2017, to June 25, 2018) and 8.28 °C, 13.11 MJ m^−2^ d^−1^, and 1.95 mm d^−1^ during the 2019 season (October 30, 2018, to June 10, 2019), respectively. The daily average mean temperature, solar radiation, and precipitation at CNU were 16.62 °C, 18.25 MJ m^−2^ d^−1^, and 3.19 mm d^−1^, respectively, during the 2018 season (February 26, 2018, to June 20, 2018).

### Proximal and RAS RS

We obtained proximally sensed data on the ground and RAS RS images at GNU in 2018 and 2019 and ground-based proximal sensing at CNU in 2018. In this study, all the field campaigns to measure the wheat canopies' reflectances were conducted an hour before or after the local solar noon (12:40 p.m. KST) to minimize perspective effects on the wheat's target sensing zone canopies or scene images. A hand-held multispectral radiometer, MSR16R (CROPSCAN, Inc., Rochester, MN, USA), was employed to measure the wheat canopy reflectance in order to define growth and development conditions during the principal development stages. MSR16R can quantify 16 wavebands within the range of 450 and 1,750 nm. The proximal sensing procedures at both GNU and CNU were performed on the same dates along with the LAI measurements to determine vegetation indices (VIs) by using canopy reflectance values from the wavebands at 800, 660, and 560 nm, as discussed in the following subsection RSCM for Wheat. A RAS, eBee (senseFly, Cheseaux-sur-Lausnne, Switzerland), was used to obtain aerial sensing images for the experimental wheat field at GNU. The RAS comprises a fixed-wing UAV (size of 960 mm and a weight of 700 g) and a digital camera, Powershot S110 NIR (Cannon, Inc., Japan), with a 12.1 MP sensor and three wavebands of green (G), red (R), and near infra-red (NIR). The center wavelengths of the bands are 550 nm (G), 625 nm (R), and 850 nm (NIR). RAS RS images were obtained on the 87, 101, 115, 130, 144, and 158 DOY in 2018 and the 53, 67, 81, 102, 123, and 144 DOY in 2019. These image data were radiometrically rectified to represent wheat growth conditions and mosaicked to deliver entire experimental field scenes using Pix4D mapper software (Pix4D S.A., Prilly, Switzerland). The processed images were then geometrically corrected using ERDAS IMAGINE software (Hexagon Geospatial, Madison, AL, USA), followed by georeferencing and registration using ArcGIS software (Esri, Inc., Redlands, CA, USA).

### RSCM for Wheat

**Figure 1:**
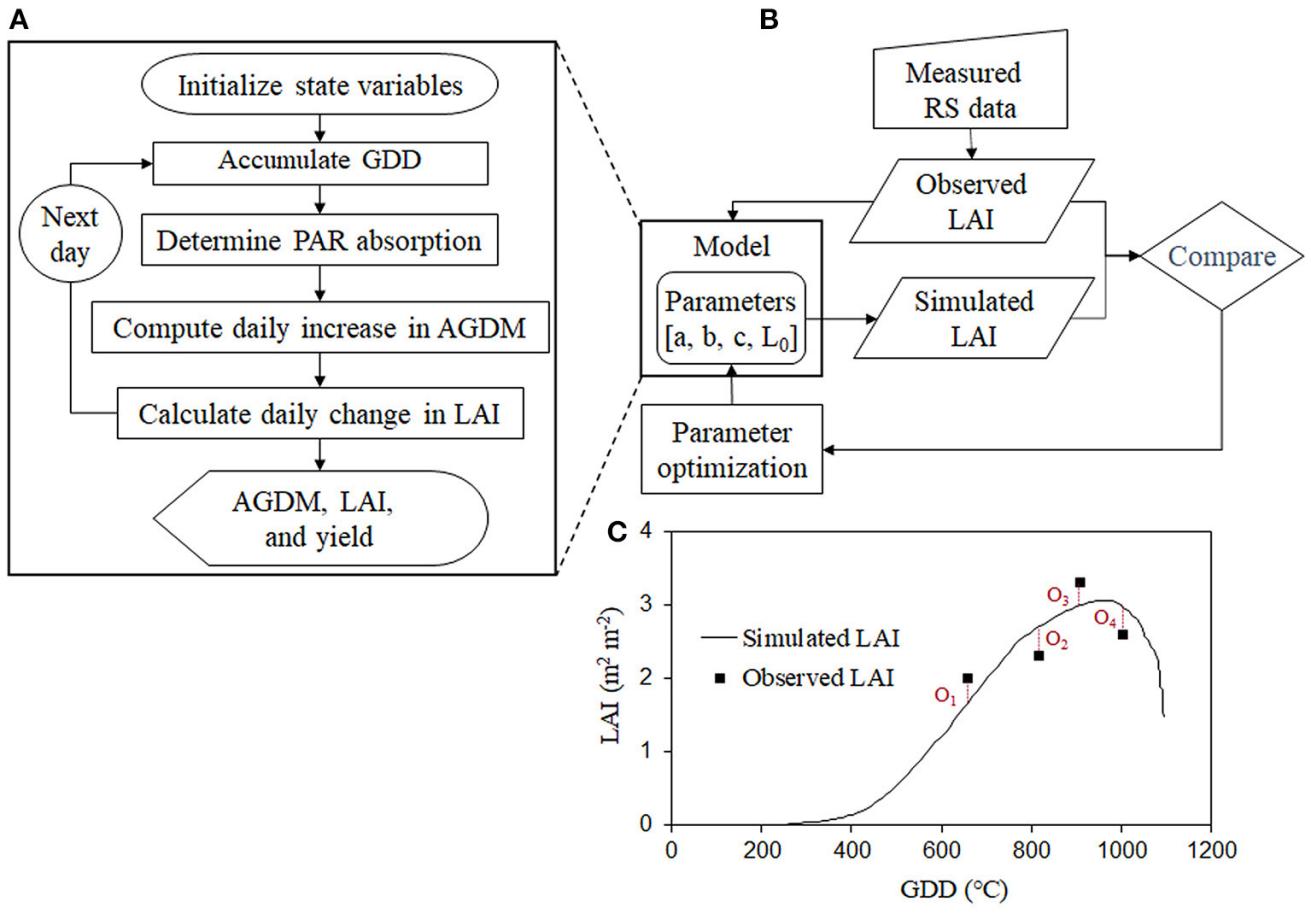
Schematic diagram of the remote sensing-integrated wheat model: **(A)** crop simulation procedure, **(B)** model parameterization based on remote sensing (RS) information, and **(C)** simulated and observed leaf area index (LAI) using the optimization process. AGDM and PAR represent above-ground dry mass and photosynthetically active radiation, respectively.

RSCM is a mathematical crop-modeling system that can simulate crop growth and yield using simple input requirements, owing to the integration of RS information (Figure 1). The RSCM for wheat simulates daily wheat growth through four simple processes, i.e., determining the daily growing degree days (GDD), the absorption of incident solar radiation by the crop canopy, the daily increase in AGDM, and the daily change in LAI (increase or senescence), based on arithmetic designs (Supplementary Table 1). RSCM is formulated such that the simulated LAI values agree with the observed LAI values, by using a Bayesian model as a part of the within-season calibration procedure. The model is coded using an IDL program version 8.5 (https://www.l3harrisgeospatial.com/Software-Technology/IDL).

We employed VIs to evaluate the wheat canopy growth based on the integrated modeling system. These included the normalized difference vegetation index (NDVI) (Rouse et al., 1974), optimized soil adjusted vegetation index (OSAVI) (Rondeaux et al., 1996), modified triangular vegetation index 1 (MTVI1) (Haboudane et al., 2004), and re-normalized difference vegetation index (RDVI) (Roujean and Breon, 1995). The four VIs were determined using the following formulas:

(1) $$NDVI=(R_{800}-R_{660})/(R_{800}+R_{660})$$

(2) $$RDVI=(R_{800}-R_{660})/\sqrt{\left(R_{800}+R_{660}\right)}$$

(3) $$OSAVI=(R_{800}-R_{660})/(R_{800}+R_{560}+0.16)$$

(4) $$MTVI1=1.2\cdot [1.2\cdot (R_{800}-R_{660})-2.5\cdot (R_{660}-R_{560})]$$

where R_800_, R_660_, and R_560_ represent the reflectance at 800, 660, and 560 nm, respectively.

As the plant canopy forms the plant's top surface, the VI, or reflectance, is most likely a two-dimensional data type representing the electromagnetic radiation exposure of the plant canopy. In contrast, LAI is a three-dimensional concept. We assumed that a log-log regression model with a slope of approximately two-thirds of the LAI could define the relationship between the VIs and the LAI. Based on this scheme, we formulated the correlations between LAI and four VIs (i.e., MTVI1, NDVI, RDVI, and OSAVI) using the following log-log linear regression model:

(5) $$\log\left(VI_{t}\right)=\alpha_{VI}+\beta_{VI}\log\left(LAI_{t}\right)+\epsilon_{t}$$

where α_*VI*_, β_*VI*_, and ϵ_*t*_ ($\sim N\left(0,\sigma_{VI}^{2}\right)$ represent the intercept, slope, and error of the log-log linear regression model, respectively. This scheme was adopted to obtain more robust LAI estimations using an ensemble method based on the relationships between the four VIs and LAI (Nguyen et al., 2019). The development of LAI for each pixel was defined by the RSCM-wheat regime by using four parameters of θ= (*L*_0_*, a, b*, and *c*). Separate parameter values were generated from the prior distribution, ψ~*N*(μ, *D*), ranging between 0 and 1 by using the following transformations:

(6) $$\begin{array}{l} \psi =\left(\psi_{1},\psi_{2},\psi_{3},\psi_{4}\right)\\ \;=\left(\log\frac{a}{1-a},\log\frac{b}{1-b},\log\frac{c}{1-c},\log\frac{L_{0}}{1-L_{0}}\;\right),\\ \;\theta =\theta \left(\psi \right)=\left(\frac{e^{\psi_{1}}}{1+e^{\psi_{1}}},\frac{e^{\psi_{2}}}{1+e^{\psi_{2}}},\frac{e^{\psi_{3}}}{1+e^{\psi_{3}}},\frac{e^{\psi_{4}}}{1+e^{\psi_{4}}}\right) \end{array}$$

We acquired both the log-log linear regression coefficients $\left(\alpha_{\ell },\beta_{\ell },\sigma_{\ell }^{2}\right),\;\ell =1,\;2,\;3,\;and\;4$, and the hyper-parameters (μ, *D*) from the data collected in this study for parameter estimation. These included the measured values of the VIs and LAI (refer to Supplementary Figure 4). Each of the parameter, μ, was specified using the “before-calibration” values (*L*_0_ = 0.2, *a* = 3.25 × 10^−1^, *b* = 1.25 × 10^−3^, and *c* = 1.25 × 10^−3^). Parameter D is a diagonal matrix with all diagonal elements equivalent to 0.5. The following arithmetic procedure was employed to obtain θ for each pixel. In Step 1, set μ served as the initial guess of ψ for each pixel. In Step 2, we define $LAI_{t}=\tilde{G}\left(t;\psi \right)=G\left(t;\theta \left(\psi \right)\right)$ and use the following objective function:

(7) $$\begin{array}{l} \sum_{\ell =1}^{5}\left\{\frac{1}{\sigma_{\ell }^{2}}\sum_{t=1}^{n}{\left(\log VI_{\ell t}-\alpha_{\ell }-\beta_{\ell }\log\tilde{G}(t;\psi )\right)}^{2}\right\}\\ +{\left(\psi -\mu \right)}^{\prime }D^{-1}\left(\psi -\mu \right) \end{array}$$

In Step 3, the simulated curve for each pixel is obtained from the estimated ψ in Step 2. In Step 4, μ, *D* is updated using the sample means and variances from the estimations in Step 2. In this calculation method, the parameter ψ was estimated by minimizing the abovementioned function. Optimization was achieved using the POWELL optimization routine (Press et al., 1992) for one-point simulation cases and the Quasi-Newton minimizer (Nash, 1990) for two-dimensional simulation cases. The POWELL optimizer is included in the IDL program. Meanwhile, we designed the Quasi-Newton minimizer callable from a separate C program to efficiently deal with big data.

The performance of the RSCM-wheat regime was evaluated using a two-sample paired *t*-test as well as two statistical indices—the root mean squared error (RMSE) and the Nash–Sutcliffe model efficiency (NSE) (Nash and Sutcliffe, 1970)—with Python (https://www.python.org). The power analysis for the *t*-test (α = 0.05) showed that power 0.5 is achievable with an effect size of 0.99 and a 9 sample size that we obtained for each element in the field measurement practice. NSE verifies the relative magnitude of the residual variance of simulated data in comparison with the observed data variance. Consequently, this index can assess how well the observed and simulated data fit the 1:1 line in a scatter plot. NSE values can range from –∞ to 1. The model is dependable if the NSE value is close to 1. In contrast, the simulated data are considered less consistent if the NSE value is nearer to zero.

## Results

### Formulation and Evaluation of RSCM for Wheat

**Figure 2:**
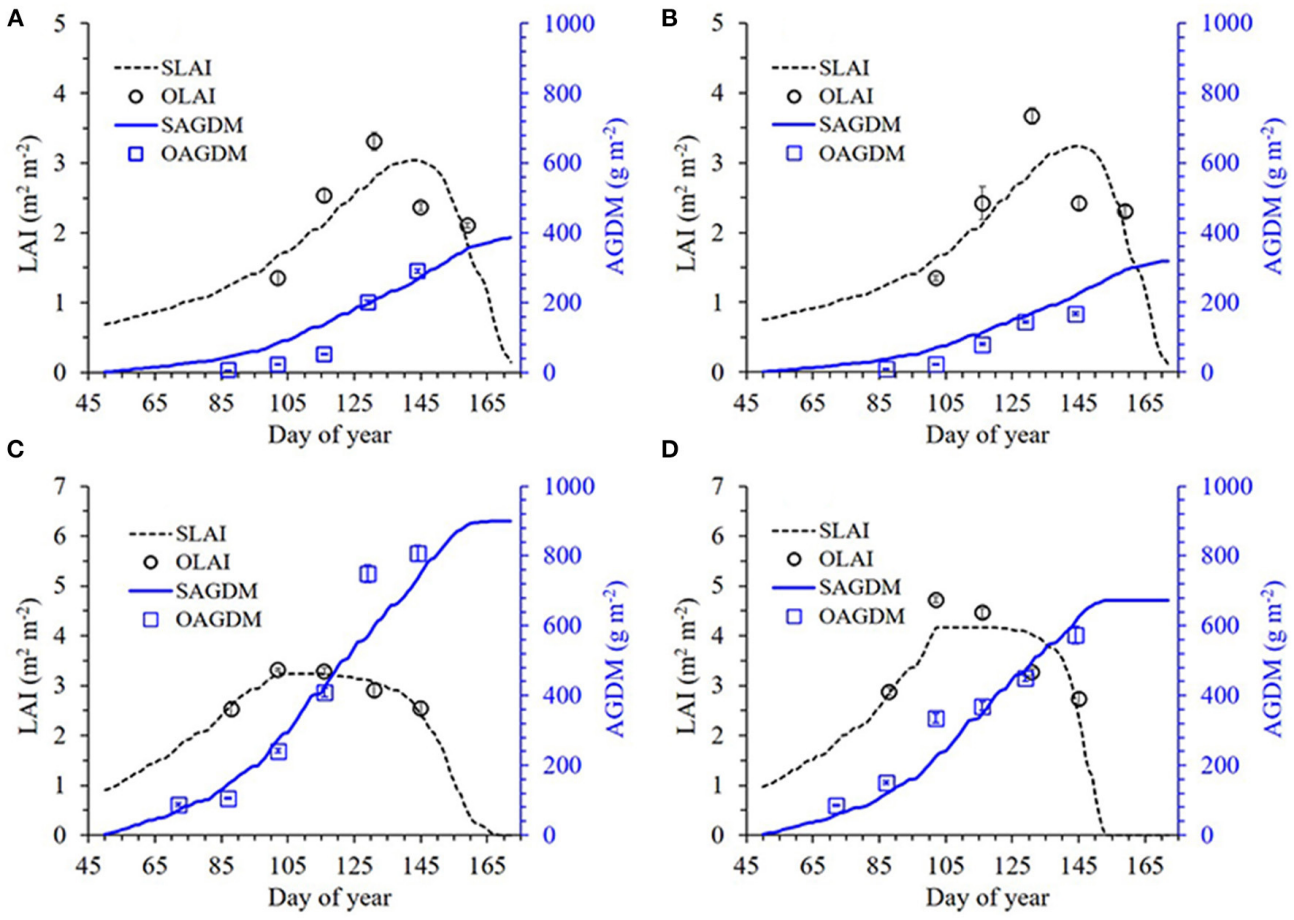
Simulated and measured leaf area index (LAI) and above-ground dry mass (AGDM) of Chokyung **(A,C)** and Keumkang **(B,D)** wheat seeded in spring **(A,B)** and fall **(C,D)** at Gyeongsang National University, Jinju, South Korea in 2018. Vertical bars represent the standard deviations of the mean values at 95% confidence intervals (*n* = 9).

We formulated the RSCM for wheat by estimating parameters specific to wheat growth from the data set obtained at GNU, Jinju, in 2018 for effective calibration of the model (refer to Supplementary Figures 1–3 and Supplementary Table 1). These parameters included radiation use efficiency (RUE), specific leaf area (SLA), and light extinction coefficient (k). The formulated modeling system was then calibrated using the dataset obtained for parameter estimation. During calibration, the simulated LAI and AGDM values were in good agreement with the measured LAI and AGDM values; the RMSE ranged from 0.11 to 0.56 m^2^ m^−2^ for LAI and from 37.9 to 82.9 g m^−2^ for AGDM, whereas the Nash–Sutcliffe efficiency (NSE) ranged from 0.10 to 0.89 for LAI and from 0.58 to 0.92 for AGDM (Figure 2 and Table 1). We found that the modeling system closely reproduced the measured data showing differences in LAI and AGDM between the cultivars and planting seasons.

**Table 1:** Comparison of root mean square error (RMSE) and Nash–Sutcliffe efficiency (NSE) between simulated (S) and measured (M) values of leaf area index (LAI) and above-ground dry mass (AGDM) of wheat cultivars grown in the spring and fall seasons of 2018 at Gyeongsang National University (GNU), Jinju, South Korea, for model calibration.

**Season**	**Cultivar**	**LAI**				**AGDM**			
		**S**	**M**	**RMSE**	**NSE**	**S**	**M**	**RMSE**	**NSE**
		**—— m**^****2****^ **m**^****−2****^ **—–**			**Unitless**	**———- g m**^****−2****^ **———**			**Unitless**
Spring	Chokyung	2.29	2.33	0.46	0.25	145.9	114.8	51.1	0.58
	Keumkang	2.38	2.43	0.56	0.10	120.0	84.8	37.9	0.65
Fall	Chokyung	2.91	2.92	0.11	0.89	370.2	400.3	82.9	0.87
	Keumkang	3.51	3.61	0.47	0.61	305.8	327.4	52.0	0.92

**Figure 3:**
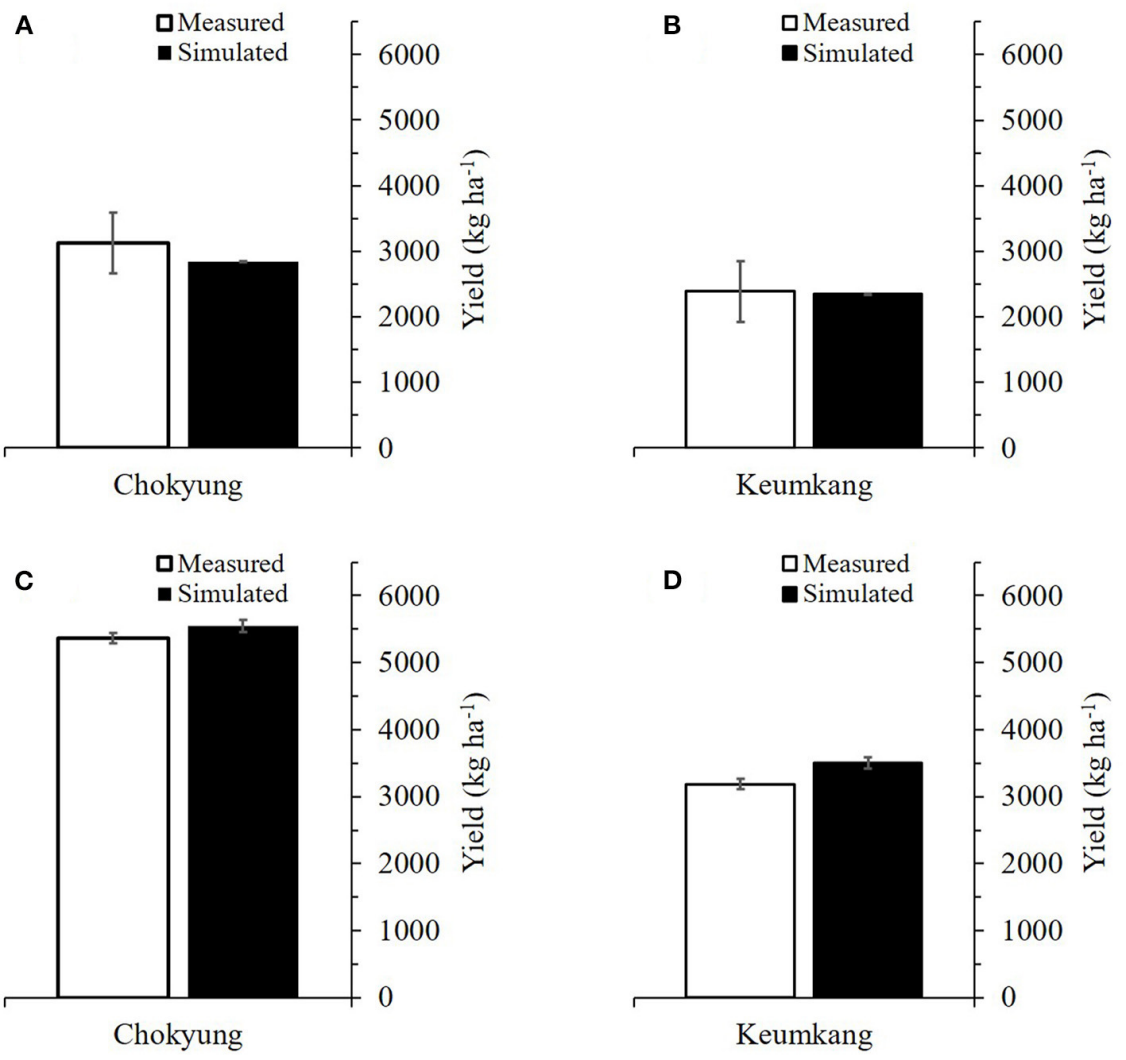
Comparison between simulated and measured grain yields of Chokyung and Keumkang wheat seeded in spring **(A,B)** and fall **(C,D)** at Gyeongsang National University, Jinju, South Korea in 2018. Vertical bars represent the standard errors of the mean yields at 95% confidence intervals (*n* = 9).

The simulated grain yields were in good agreement with the measured yields, with the RMSE ranging from 0.208 to 0.942 ton ha^−1^ and without any significant differences (a range of *p* from 0.273 to 0.905), as indicated by the two-sample *t-*tests (α = 0.05) (Figure 3 and Table 2). Likewise, the RSCM system reproduced the measured value differences in grain yield between the cultivars and planting seasons.

**Table 2:** Comparison of root mean square error (RMSE) and *p* according to two-sample *t-*tests between simulated (S) and measured (M) yields of wheat cultivars grown in the spring and fall seasons of 2018 at Gyeongsang National University (GNU), Jinju, South Korea, for model calibration.

**Season**	**Cultivar**	**Simulated**	**Measured**	**RMSE**	***p* (α = 0.05)**
		**—————- ton ha**^****−1****^ **—————-**			**Unitless**
Spring	Chokyung	2.842	3.124	0.846	0.645
	Keumkang	2.349	2.389	0.451	0.905
Fall	Chokyung	5.549	5.370	0.208	0.273
	Keumkang	3.501	3.189	0.942	0.656

**Figure 4:**
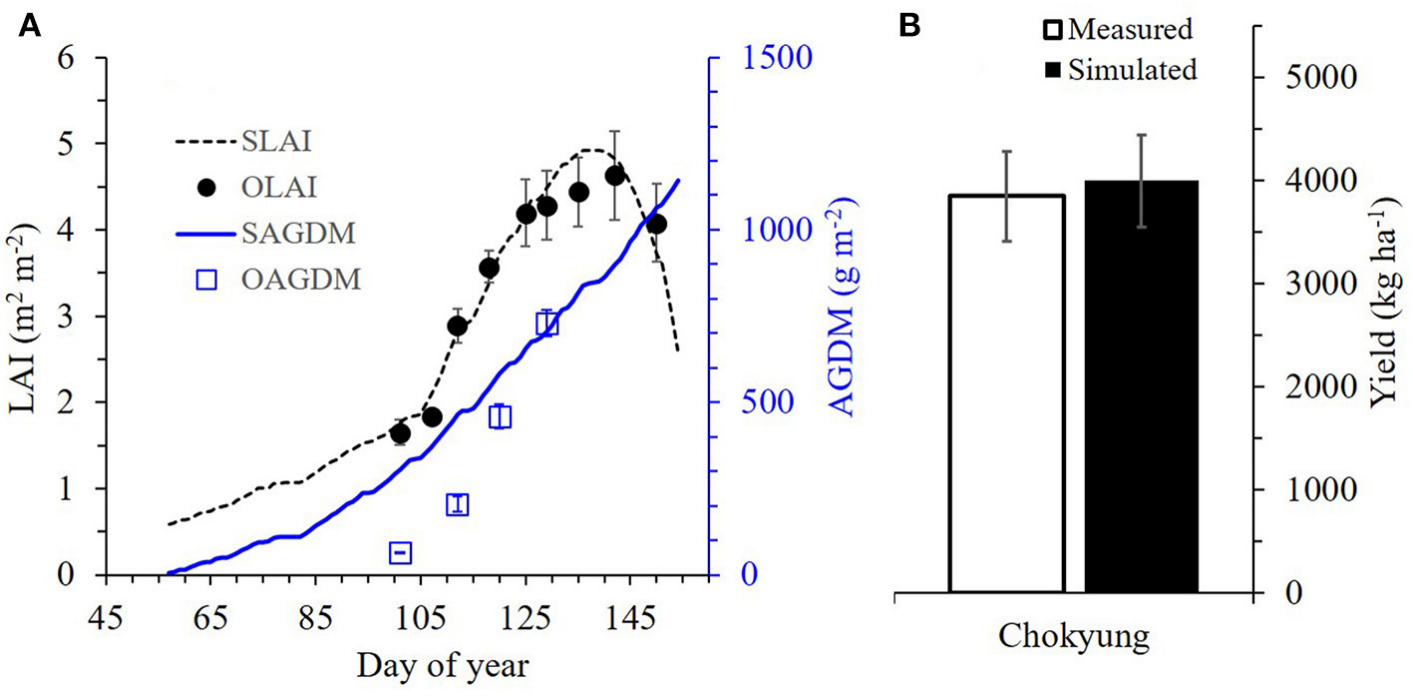
Simulated and measured leaf area index (LAI) and above-ground dry mass (AGDM) vs. measured LAI and AGDM of Chokyung wheat at Chonnam National University, Gwangju, South Korea, in 2018. Vertical bars represent the standard deviations **(A)** and standard errors **(B)** of the mean values at 95% confidence intervals (*n* = 9).

**Figure 5:**
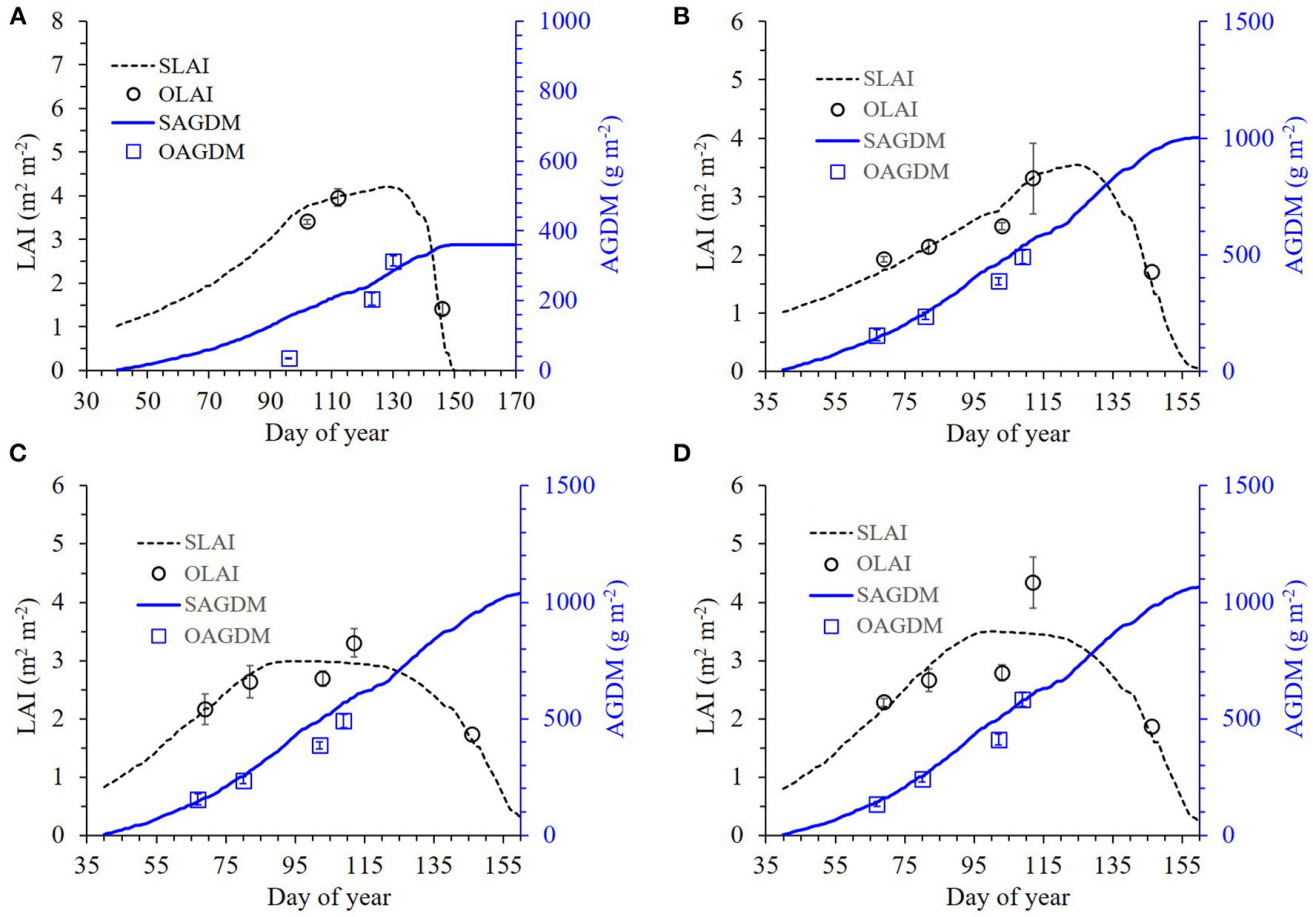
Simulated and measured leaf area index (LAI) and above-ground dry mass (AGDM) of Chokyung wheat seeded in spring **(A)** and fall with different nitrogen applications of 40 kg ha^−1^ at planting, 30 kg ha^−1^ at rejuvenation, and 0 kg ha^−1^ at initial reproduction (N40-30-0) **(B)**, N40-30-30 **(C)**, and N40-30-60 **(D)** at Gyeongsang National University, Jinju, South Korea, in 2019. Vertical bars represent the standard deviations of the mean values at 95% confidence intervals (*n* = 9).

The wheat-modeling regime was then validated for its consistency by using datasets separately obtained during the wheat-growing seasons at both CNU in 2018 and GNU in 2019. During validation using the dataset in 2018 from CNU, the simulated LAI and AGDM values agreed with the corresponding measured values, with RMSEs of 0.24 m^2^ m^−2^ for LAI and 187.8 g m^−2^ for AGDM and NSEs of 0.95 for LAI and −0.61 for AGDM (Figure 4 and Table 3). There was no significant difference between the simulated and measured grain yields (*p* = 0.858), according to a two-sample *t-*test (α = 0.05), with an RMSE of 1.512 ton ha^−1^ (Table 4). At GNU, for the wheat season of 2019, the simulated LAI and AGDM values corresponded to the measured LAI and AGDM values, with the RMSE ranging from 0.19 to 0.52 m^2^ m^−2^ for LAI and from 44.2 to 75.5 g m^−2^ for AGDM and the NSE ranging from 0.44 to 0.92 for LAI and from −0.93 to 0.94 for AGDM (Figure 5 and Table 3). The RSCM system closely simulated the measured differences in LAI and AGDM between the regions and nitrogen application regimes.

**Table 3:** Comparison of root mean square error (RMSE) and Nash–Sutcliffe efficiency (NSE) between simulated (S) and measured (M) values of leaf area index (LAI) and above-ground dry mass (AGDM) of Chokyung wheat seeded in the fall of 2018 at CNU, Gwangju, and seeded in the spring and fall seasons of 2019 at Gyeongsang National University (GNU), Jinju, south Gyeongsang province, South Korea, with different amounts of nitrogen (N) applications of 40 kg ha^−1^ at planting, 30 kg ha^−1^ at rejuvenation, and 0 kg ha^−1^ at initial reproduction (N40-30-0), N40-30-30, and N40-30-60 for model validation.

**Season and site**	**N treatment**	**LAI**				**AGDM**			
		**S**	**M**	**RMSE**	**NSE**	**S**	**M**	**RMSE**	**NSE**
		**—— m**^****2****^ **m**^****−2****^ **—–**			**Unitless**	**———- g m**^****−2****^ **———**			**Unitless**
Fall, CNU	40-30-30	3.57	3.51	0.24	0.95	514.7	364.7	187.8	−0.61
Spring, GNU	40-30-30	2.90	2.94	0.44	0.90	229.3	184.6	75.5	−0.93
Fall, GNU	40-30-0	2.31	2.32	0.19	0.92	346.8	316.7	44.7	0.92
	40-30-30	2.50	2.50	0.22	0.83	363.9	316.7	66.0	0.85
	40-30-60	2.76	2.79	0.52	0.44	367.7	341.8	44.2	0.94

**Table 4:** Comparison of root mean square error (RMSE) and *p* according to two-sample *t-*tests between simulated (S) and measured (M) yields of Chokyung wheat seeded in the fall of 2018 at Chonnam National University (CNU), Gwangju, and seeded in the spring and fall seasons of 2019 at Gyeongsang National University (GNU), Jinju, south Gyeongsang province, South Korea, with different amounts of nitrogen (N) applications of 40 kg ha^−1^ at planting, 30 kg ha^−1^ at rejuvenation, and 0 kg ha^−1^ at initial reproduction (N40-30-0), N40-30-30, and N40-30-60 for model validation.

**Season and site**	**N treatment**	**Simulated**	**Measured**	**RMSE**	***p* (α = 0.05)**
		**—————- ton ha**^****−1****^ **—————-**			**Unitless**
Fall, CNU	40-30-30	3.991	3.843	1.512	0.858
Spring, GNU	40-30-30	6.445	6.513	0.438	0.276
Fall, GNU	40-30-0	3.050	3.317	1.175	0.717
	40-30-30	4.561	4.842	0.728	0.389
	40-30-60	4.067	5.066	1.456	0.859

**Figure 6:**
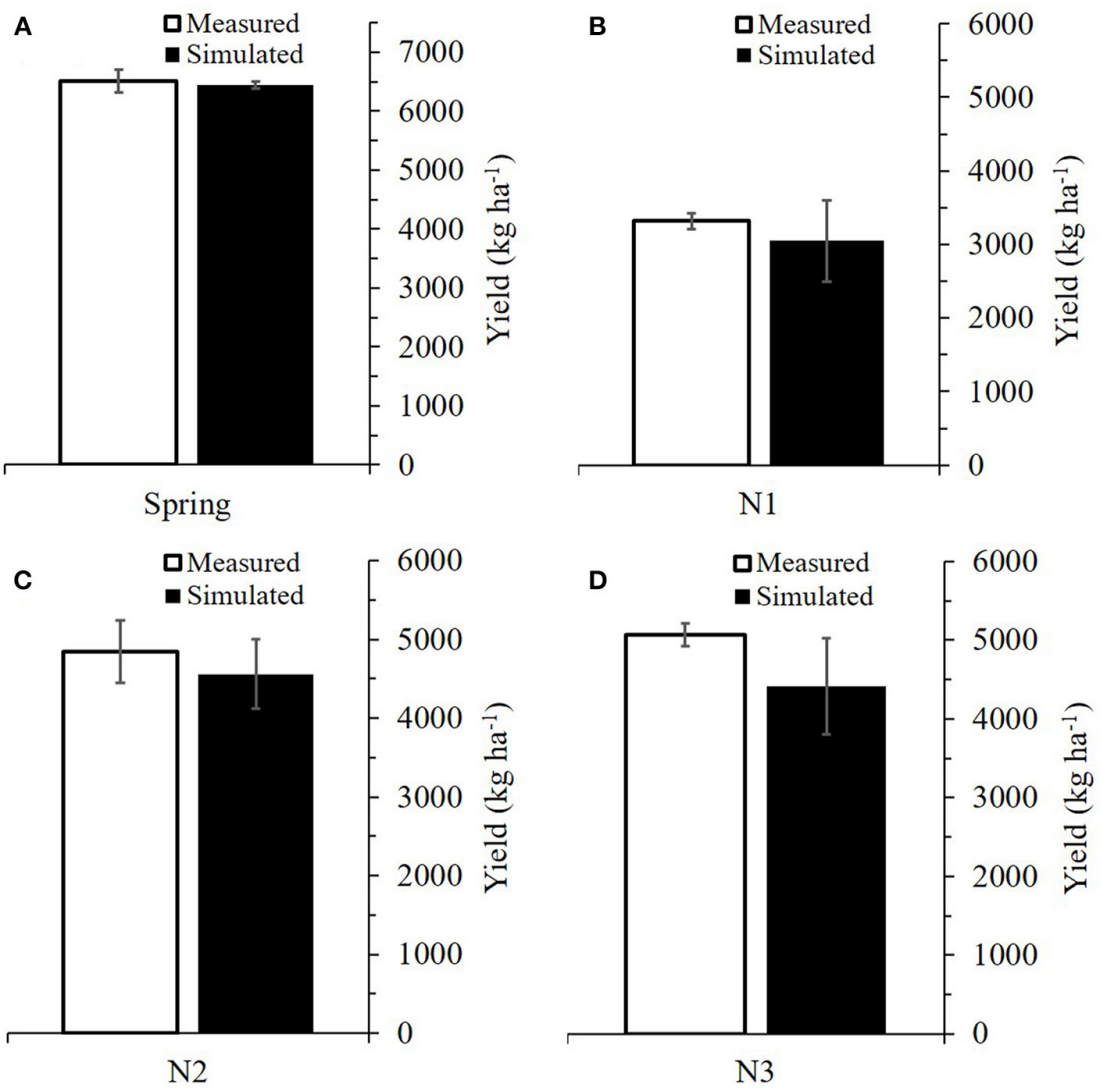
Comparison between simulated and measured grain yields of Chokyung wheat seeded in spring **(A)** and fall with different nitrogen applications of 40 kg ha^−1^ at planting, 30 kg ha^−1^ at rejuvenation, and 0 kg ha^−1^ at initial reproduction (N40-30-0) **(B)**, N40-30-30 **(C)**, and N40-30-60 **(D)** at Gyeongsang National University, Jinju, South Korea, in 2019. Vertical bars represent the standard errors of the mean values at 95% confidence intervals (*n* = 9).

There were no significant differences between the simulated and measured grain yields (with *p* ranging from 0.276 to 0.859), according to two-sample *t-*tests (α = 0.05); furthermore, the RMSE ranged from 0.438 to 1.456 ton ha^−1^ (Figure 6 and Table 4). The modeling system also simulated the measured grain yield differences between the regions and nitrogen application regimes.

### Two-Dimensional Simulation of Wheat

**Figure 7:**
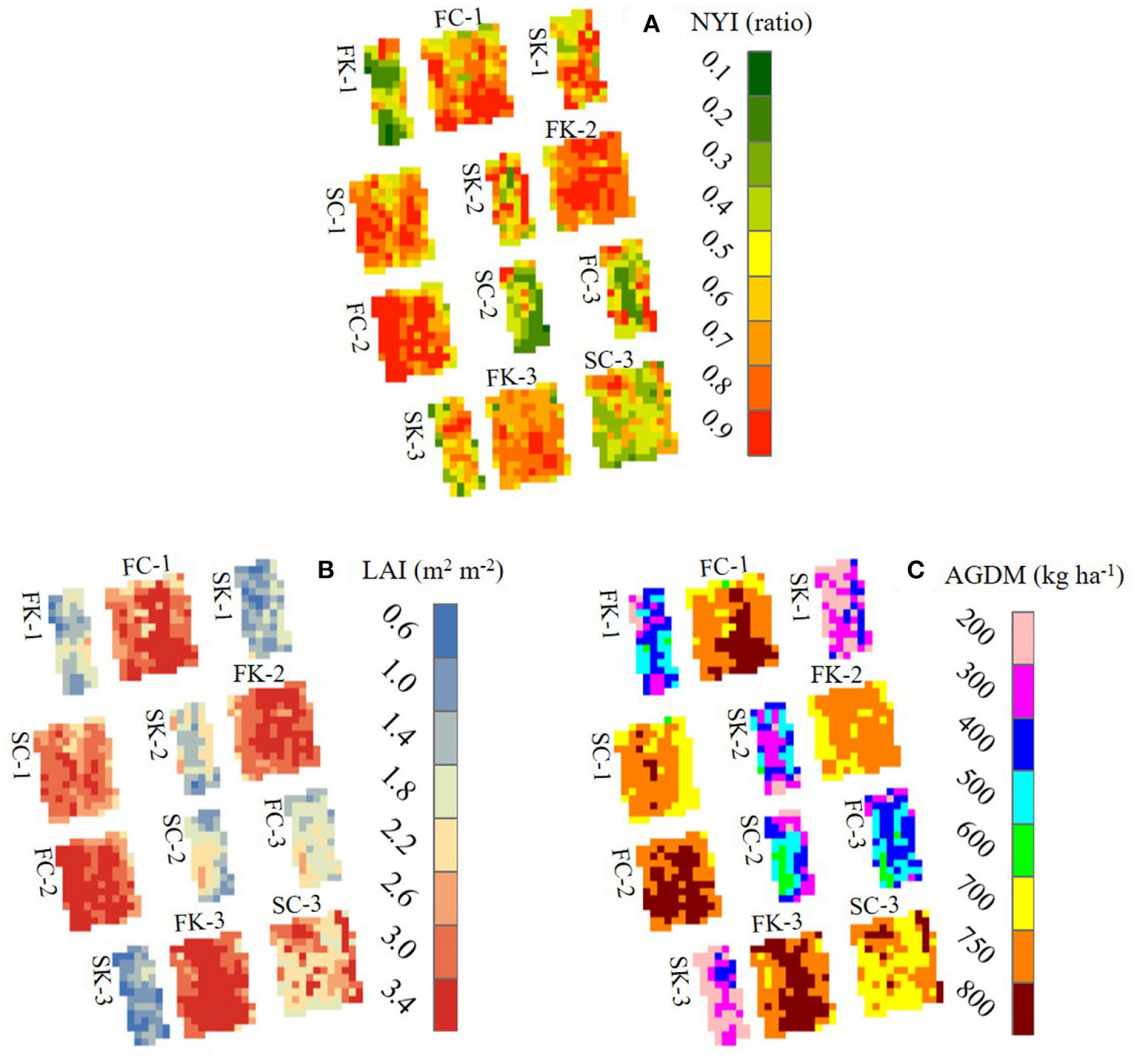
Two-dimensional simulated projections of normalized yield index, NYI **(A)**, leaf area index, LAI **(B)**, and above-ground dry mass, AGDM **(C)** of two wheat cultivars seeded in fall and spring 2018 at Gyeongsang National University, Jinju, South Korea. The remote-controlled aerial image data for **(B,C)** were obtained 60 days after rejuvenation. FC, fall-seeded Chokyeong; FK, fall-seeded Keumkang; SC, spring-seeded Chokyeong; SK, spring-seeded Keumkang; the numbers after each upper character and dash symbol represent experimental blocks.

**Figure 8:**
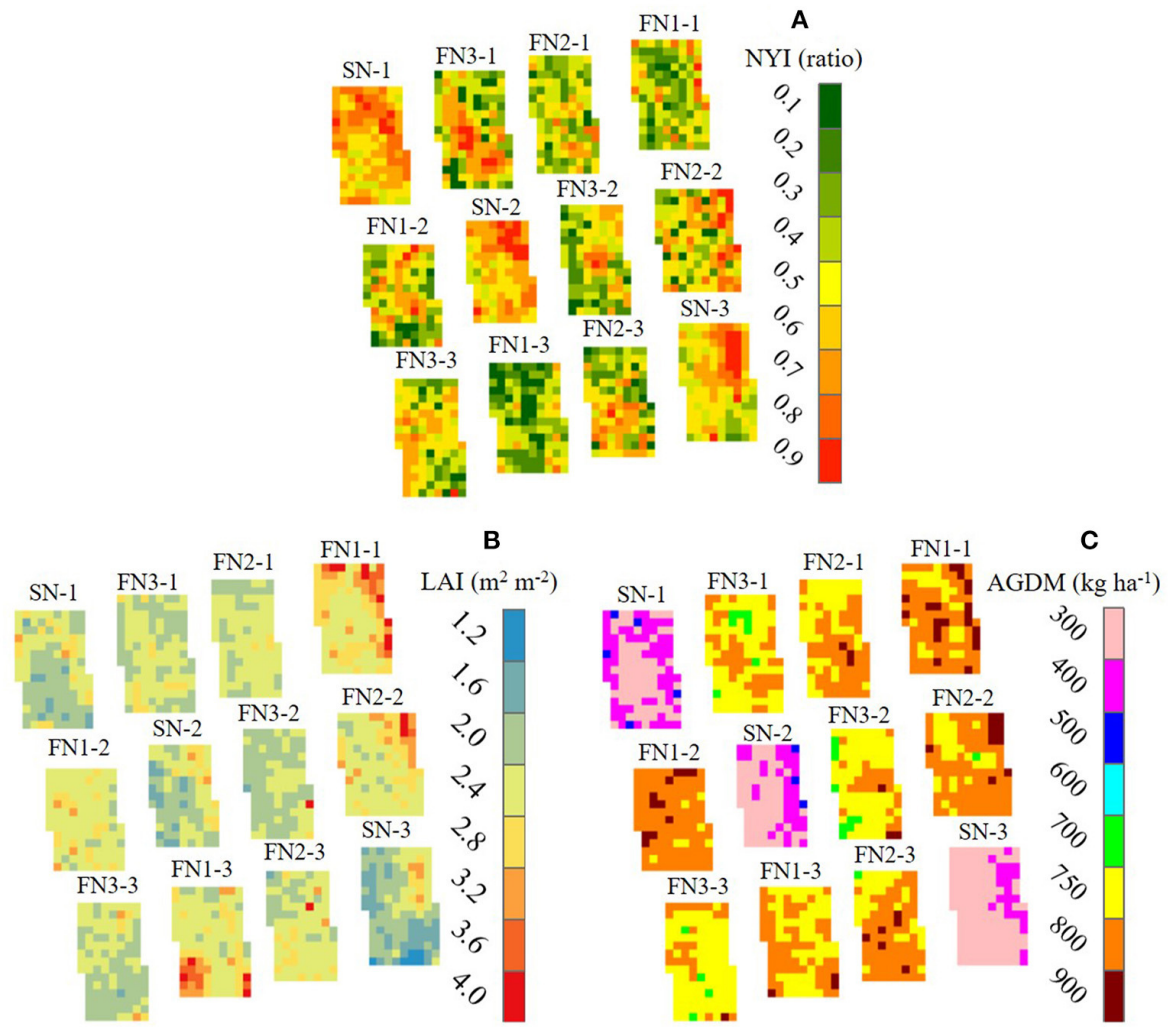
Two-dimensional simulated projections of normalized yield index, NYI **(A)**, leaf area index, LAI **(B)**, and above-ground dry mass, AGDM **(C)** of Chokyeong wheat treated with different nitrogen (N) levels at the tillering and heading stages at Gyeongsang National University, Jinju, South Korea, in 2019. The remote-controlled aerial image data for **(B,C)** were obtained 60 days after rejuvenation. FN1, fall-seeded with different nitrogen applications of 40 kg ha^−1^ at planting, 30 kg ha^−1^ at rejuvenation, and 0 kg ha^−1^ at initial reproduction (N40-30-0); FN2, fall-seeded N40-30-30; FN3, fall-seeded N40-30-60; SN, spring-seeded N40-30-30; the numbers after each dash symbol represent experimental blocks.

The RSCM for wheat was applied for reproducing two-dimensional wheat growth and yield variations for the datasets obtained at GNU in 2018 and 2019 (Figures 7, 8). The RSCM system closely simulated the field variabilities of normalized yield index (NYI), LAI, and AGDM due to different planting regimes and cultivars in the wheat season of 2018 (Figure 7). The simulated NYI, LAI, and AGDM were larger for the fall-sown wheat than the spring-sown wheat, indicating comparatively greater variability in the spring-seeded wheat (Table 5). The RSCM system also presented apparent seasonal growth differences in terms of LAI and AGDM because of the differences in seasonal planting periods.

**Table 5:** Descriptive statistical indices (DSI) of mean with standard deviation (SD), maximum, and minimum for two-dimensional variation in simulated values of normalized yield index (NYI), leaf area index (LAI), and above-ground dry mass (AGDM) of wheat cultivars grown in the spring and fall seasons of 2018 at Gyeongsang National University (GNU), Jinju, South Korea.

**Season**	**Cultivar**	**DSI**	**NYI**	**LAI**	**AGDM**
			**Unitless**	**−− m^**2**^ m^**−2**^ −−**	**−− g m^**−2**^ −−**
Spring	Chokyung	Mean ± SD	0.56 ± 0.241	1.5 ± 0.40	357.1 ± 95.98
		Max	1.00	2.5	582.3
		Min	0.00	0.4	82.7
	Keumkang	Mean ± SD	0.59 ± 0.226	1.1 ± 0.46	252.5 ± 117.83
		Max	0.99	2.3	557.3
		Min	0.02	0.1	22.7
Fall	Chokyung	Mean ± SD	0.76 ± 0.085	2.9 ± 0.33	719.6 ± 31.89
		Max	0.89	3.5	785.2
		Min	0.29	1.6	586.0
	Keumkang	Mean ± SD	0.70 ± 0.163	2.7 ± 0.55	726.5 ± 36.95
		Max	0.95	3.7	785.9
		Min	0.24	1.5	595.7

Likewise, the RSCM closely simulated field variabilities in NYI, LAI, and AGDM due to the different planting regimes and N treatments for the wheat season of 2019 (Figure 8). The simulated NYI values were 0.49 ± 0.095 for the spring-sown wheat and ranged from 0.35 ± 0.116 to 0.40 ± 0.131 for the N treatments in the case of the fall-sown wheat, indicating similar variation trends of LAI and AGDM with respect to the treatment effects on the NYI (Table 6). Furthermore, it was also noted that the overall two-dimensional projection generally matched the measured data in the field presented in the earlier subsection Formulation and Evaluation of RSCM for Wheat (refer to Figures 4–6).

**Table 6:** Descriptive statistical indices (DSI) of mean with standard deviation (SD), maximum, and minimum for two-dimensional variation in simulated values of normalized yield index (NYI), leaf area index (LAI), and above-ground dry mass (AGDM) of wheat grown in the spring and fall seasons of 2019 with different nitrogen (N) treatments at Gyeongsang National University (GNU), Jinju, South Korea.

**Season**	**N treatment^a^**	**DSI**	**NYI**	**LAI**	**AGDM**
			**unitless**	**−− m^**2**^ m^**−2**^ −−**	**−− g m^**−2**^ −−**
Spring	N40-30-30	Mean ± SD	0.49 ± 0.095	1.9 ± 0.39	281.4 ± 53.21
		Max	1.00	3.1	439.0
		Min	0.27	1.1	150.5
Fall	N40-30-0	Mean ± SD	0.35 ± 0.116	2.4 ± 0.46	749.1 ± 25.40
		Max	0.74	5.1	854.8
		Min	0.09	1.9	701.0
	N40-30-30	Mean ± SD	0.40 ± 0.125	2.2 ± 0.32	761.3 ± 25.64
		Max	0.74	4.0	851.0
		Min	0.06	1.5	683.0
	N40-30-60	Mean ± SD	0.40 ± 0.131	2.0 ± 0.27	737.6 ± 24.27
		Max	0.74	5.4	847.6
		Min	0.02	1.5	649.3

a *N40-30-0, N40-30-30, and N40-30-60 indicate N applications of 40 kg ha^−1^ at planting, 30 kg ha^−1^ at rejuvenation, and 0 or 30 or 60 kg ha^−1^ at initial reproduction*.

**Figure 9:**
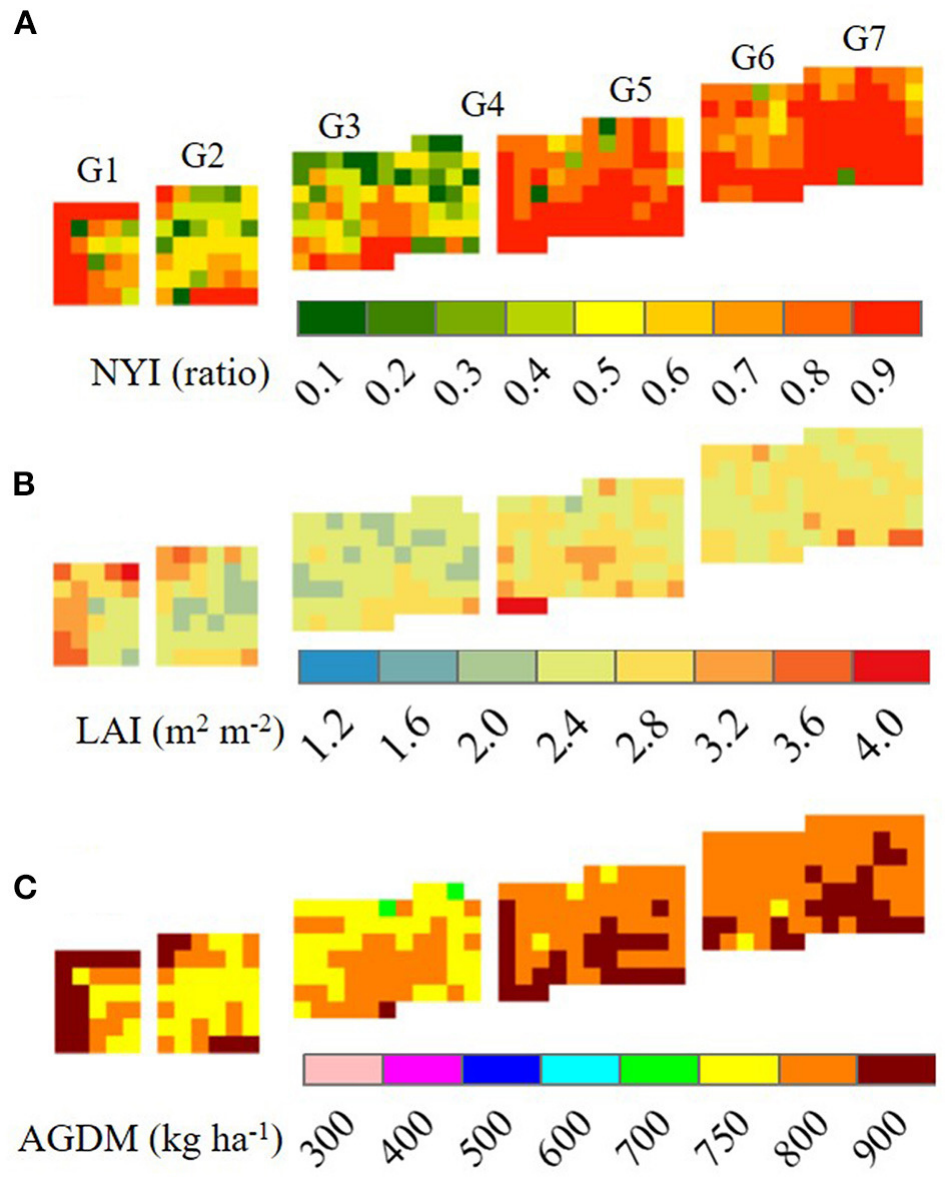
Two-dimensional simulated projections of normalized yield index, NYI **(A)**, leaf area index, LAI **(B)**, and above-ground dry mass, AGDM **(C)** of Chokyeong wheat treated with different nitrogen gradient (G) levels at the tillering and heading stages at Gyeongsang National University, Jinju, South Korea, in 2019. The remote-controlled aerial image data for **(B,C)** were obtained 60 days after rejuvenation. G1, nitrogen applications of 40 kg ha^−1^ at planting, 0 kg ha^−1^ at rejuvenation, and 0 kg ha^−1^ at initial reproduction (40-0-0); G2, 40-10-10; G3, 40-20-20; G4, 40-30-30; G5, 40-40-40-40; G6, 40-50-50; G7, 40-60-60.

We also note that the RSCM could reproduce yield and growth changes in response to different N gradient levels (Figure 9 and Supplementary Table 4). The border effects could also be observed for low levels of N (i.e., G1–G3 in Figure 9). The yields and growth conditions in the border pixels appear to have more productive environments than those in the inner pixels due to lower plant-to-plant compatibility. The spatial variations in the projected variables showed more variability under lower N levels; this is likely because, under these N levels, wheat growth depended on the variability in soil fertility rather than N fertilization.

## Discussion

This study evaluated and applied a crop-modeling system integrated with proximal sensing information for reproducing the two-dimensional variation in wheat productivity. The proposed RSCM for wheat was first calibrated based on parameter estimation of wheat growth-specific parameters (i.e., RUE, SLA, and k) by using the Jinju dataset of 2018. Previous studies have introduced parameter estimation techniques for crop simulation modeling in order to reduce the model's complexity and investigate the best possible fit (Maas, 1993a; Ahuja et al., 2000). RUE, defined as the amount of dry biomass produced per unit of intercepted solar radiation (Monteith, 1977), is an important parameter employed in many crop models (Jones et al., 2003; Ko et al., 2010; Nguyen et al., 2019). Both k and SLA are also crucial parameters in simulating crop growth and development for most RUE-based crop models (Shawon et al., 2020a). The parameter k, which is dependent on the type of crop and the leaf angle distribution, among other factors, is utilized to simulate the amount of PAR intercepted in the crop canopy (Charles-Edwards et al., 1986; Goudriaan, 1988). SLA, adopted as a concept in the analysis of whole plant growth (Gunn et al., 1999), plays a key role in determining crop productivity; this is because changes in the SLA reflect the changes in the structure and nutritional content of leaves (Gong and Gao, 2019). A crop model is generally designed to simulate crop responses to environments, considering ideal growth and management practices (Lövenstein et al., 1992). The current modeling system was also formulated and parameterized based on this strategy, by using the abovementioned growth-specific parameters.

An appropriately calibrated crop model can accurately simulate crop growth and productivity, as well as environmental conditions such as soil moisture (Ahuja et al., 2000). This could be realized through the calibration and validation results of the proposed RSCM for wheat growth and yield. Although there were a few anomalies in the statistical comparison index (i.e., NSE) for AGDM during validation at CNU and GNU, the other statistical analysis cases for AGDM indicated significant agreement between the simulated and measured values. The proposed RSCM for wheat also reproduced LAI and yield values for the different cultivars and management practices of planting and N treatments in statistically significant agreement with the corresponding measured values. Therefore, the wheat-modeling system showed potential for application in simulation case studies for different cultivation and management regimes such as planting and N treatments and the geospatial variations in growth and productivity by employing pixel-based two-dimensional simulations.

The RSCM was constructed to simulate crop growth and productivity with simple input prerequisites to integrate proximal or RS data. There have been previous endeavors to establish such integrated crop-modeling systems for barley (Shawon et al., 2020b), rice (Ko et al., 2015; Nguyen et al., 2019), and soybean (Shawon et al., 2020a). The proposed RSCM with the incorporated modeling procedure could simulate wheat growth and productivity, with statistically good precision. It would be significantly advantageous if the RSCM becomes capable of simulating major agronomic crops through the integration of RS information from various platforms, such as optical satellites (Yeom et al., 2018; Jeong et al., 2020) and remote-controlled aerial systems (Jeong et al., 2018a). The current study introduces one such prospect of applying the RSCM for wheat growth and yield monitoring by using a RAS.

The current RSCM system employing RAS-based imagery requires well-quantified proximal sensing images for enhanced applicability and distinctive images embodying the growth of the crop of interest during its growing season (Ko et al., 2015; Jeong et al., 2016). An essential enhancement is the use of radiometrically well-calibrated imagery complemented with a relatively sophisticated sensor. Enhanced RAS images could afford improved applicability of the model for monitoring crop growth conditions and productivity (Jeong et al., 2018a). Such improvements would allow the incorporation of additional values in the RSCM, thereby facilitating the investigation of agricultural production systems. The RSCM can possibly be extended and applied to information delivery systems and decision support tools for different crop cultivation management measures and practical cultivation management tools; this would afford more precise agricultural practices owing to the accurate information regarding crop growth and development conditions provided by the RAS images. Future improvements in the modeling system could involve formulating it with forecast feasibility within the crop growing season and for long periods. Such an enhanced modeling system would be more applicable as a decision-making system for various practical management scenarios.

## Conclusion

This study introduced an RSCM for simulating the field-based geospatial variations in wheat growth and yield to determine a best management practice to achieve improved wheat productivity. We assume that the modeling system is applicable for scouting wheat growth and evaluating the productivity, as well as for field management, owing to the integration with proximal or RS information from different platforms (such as sensors on the ground or aboard satellites and human- or remotely controlled aerial systems). The current study demonstrated that the RSCM system could reproduce wheat productivity with different cultivars and various nitrogen application regimes and its geospatial variation. As an advantage, a user can operate the RSCM, which has simple input requirements, with minimal climate data pertaining to solar radiation and temperatures and proximal or RS images; this is possible owing to the integration of the crop modeling scheme and sensing information. The proposed RSCM for wheat requires appropriately quantified sensing data or imagery for being applicable in more accurate productivity monitoring of wheat and different field management decisions.

## Data Availability Statement

The raw data supporting the conclusions of this article will be made available by the authors, without undue reservation. The model program code can be shared upon viable request.

## Author Contributions

JK, KL, and SS designed the research. JK wrote the manuscript with contributions from SJ and TS. AS, TS, and SJ performed the study. All authors contributed to the article and approved the submitted version.

## Conflict of Interest

The authors declare that the research was conducted in the absence of any commercial or financial relationships that could be construed as a potential conflict of interest.
