# Supplementary material for: Simulation of Wheat Productivity Using a Model Integrated With Proximal and Remotely Controlled Aerial Sensing Information
Source: Front Plant Sci. 2021 Mar 24;12:649660. doi: 10.3389/fpls.2021.649660 (PMC8024651; doi:10.3389/fpls.2021.649660)
Supplement: Supplementary file 1 [file Data_Sheet_1.pdf]

Supplementary Material

1 Supplementary Figures

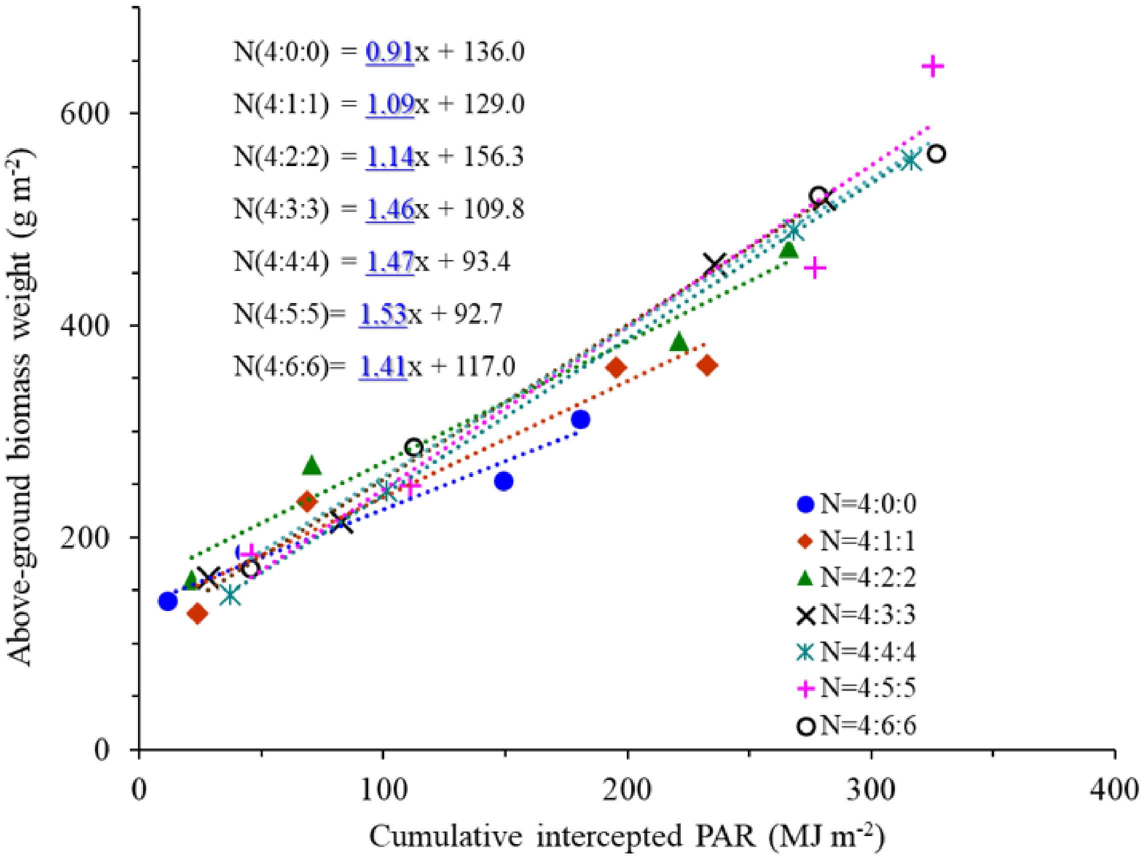

Annex Figure 1. Determination of radiation use efficiency (ε) from the slope of the regression line between amounts of accumulated photosynthetically active radiation (PAR) absorbed by barley canopies and the above-ground dry mass in 2018.

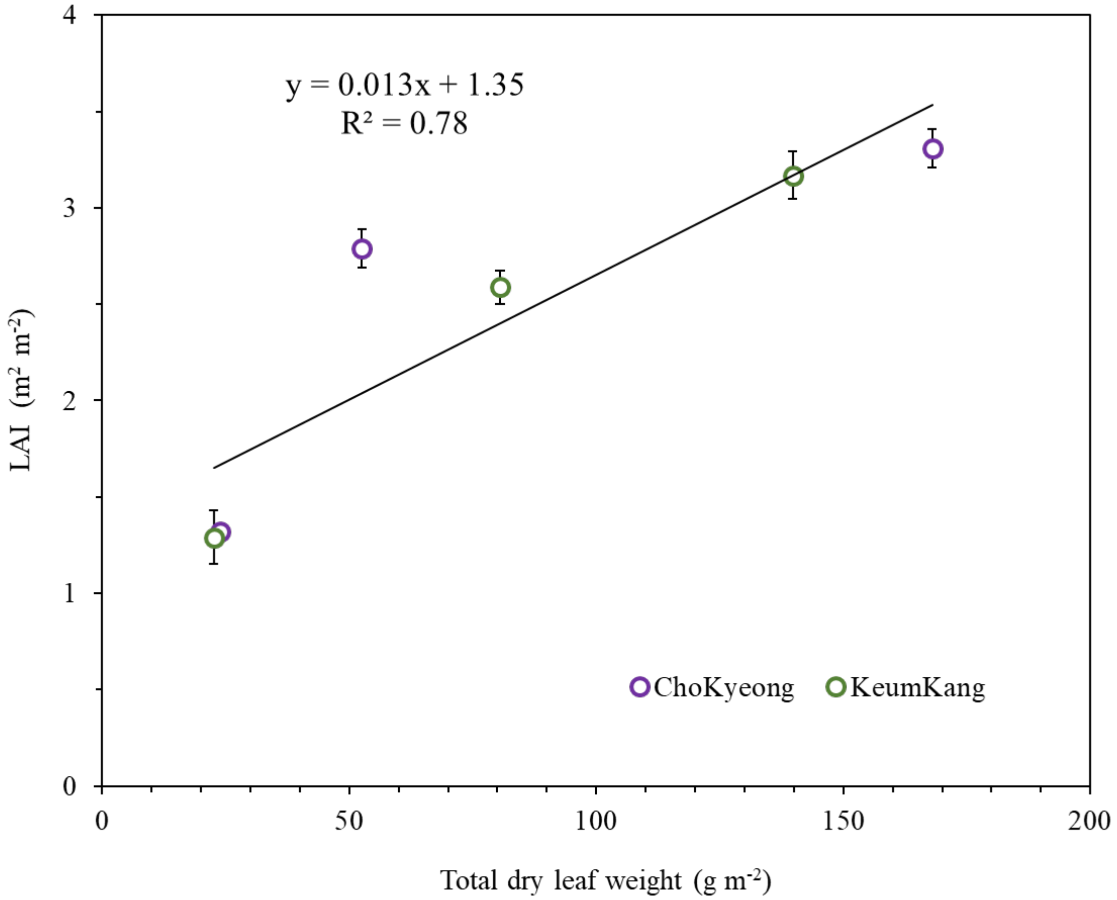

Annex Figure 2. Linear relationship between leaf area index (LAI) and leaf dry weight for five barley cultivars. The slope of the linear regression expresses the value of specific leaf area (SLA), i.e., 0.013 m^2 g^{-1}.

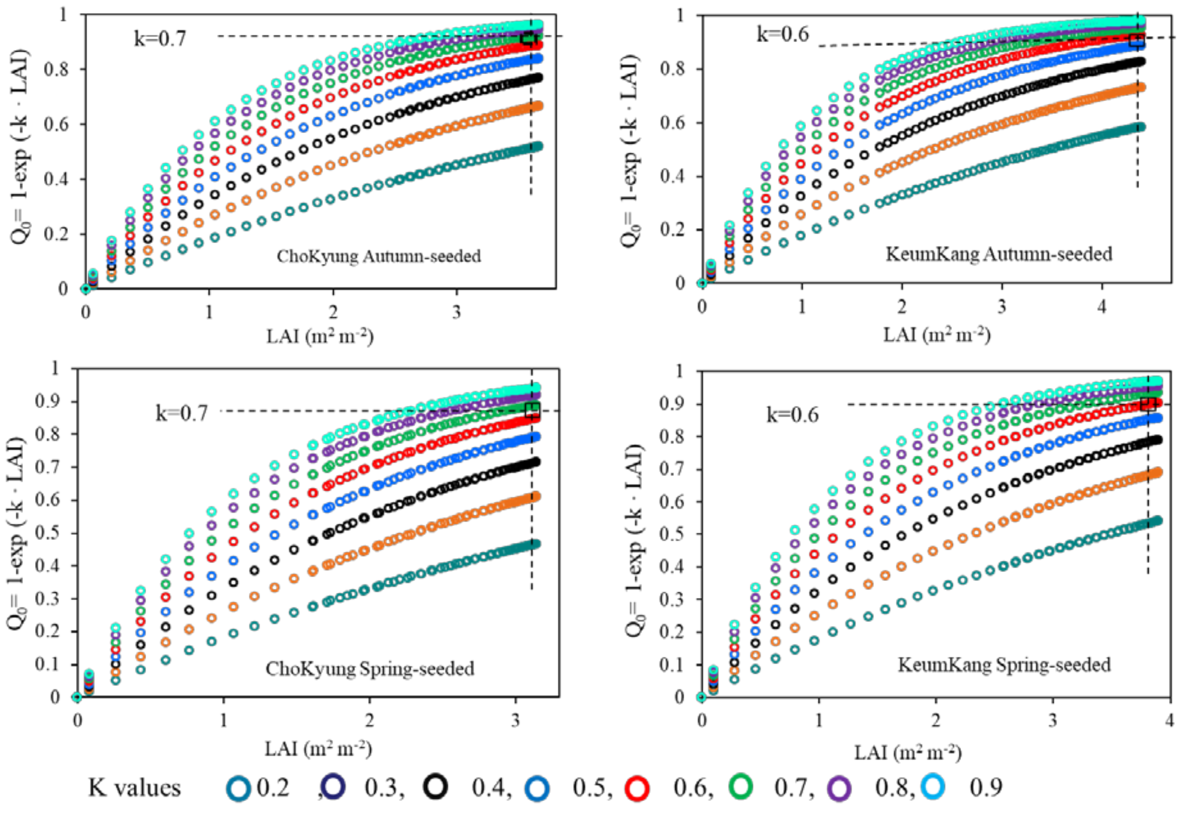

Annex Figure 3. Determination of light extinction coefficient (k) according to the relationship between canopy light absorption ($1\text{-}e^{-k \cdot LAI}$) and leaf area index (LAI) for two spring- and autumn-seeded wheat cultivars.

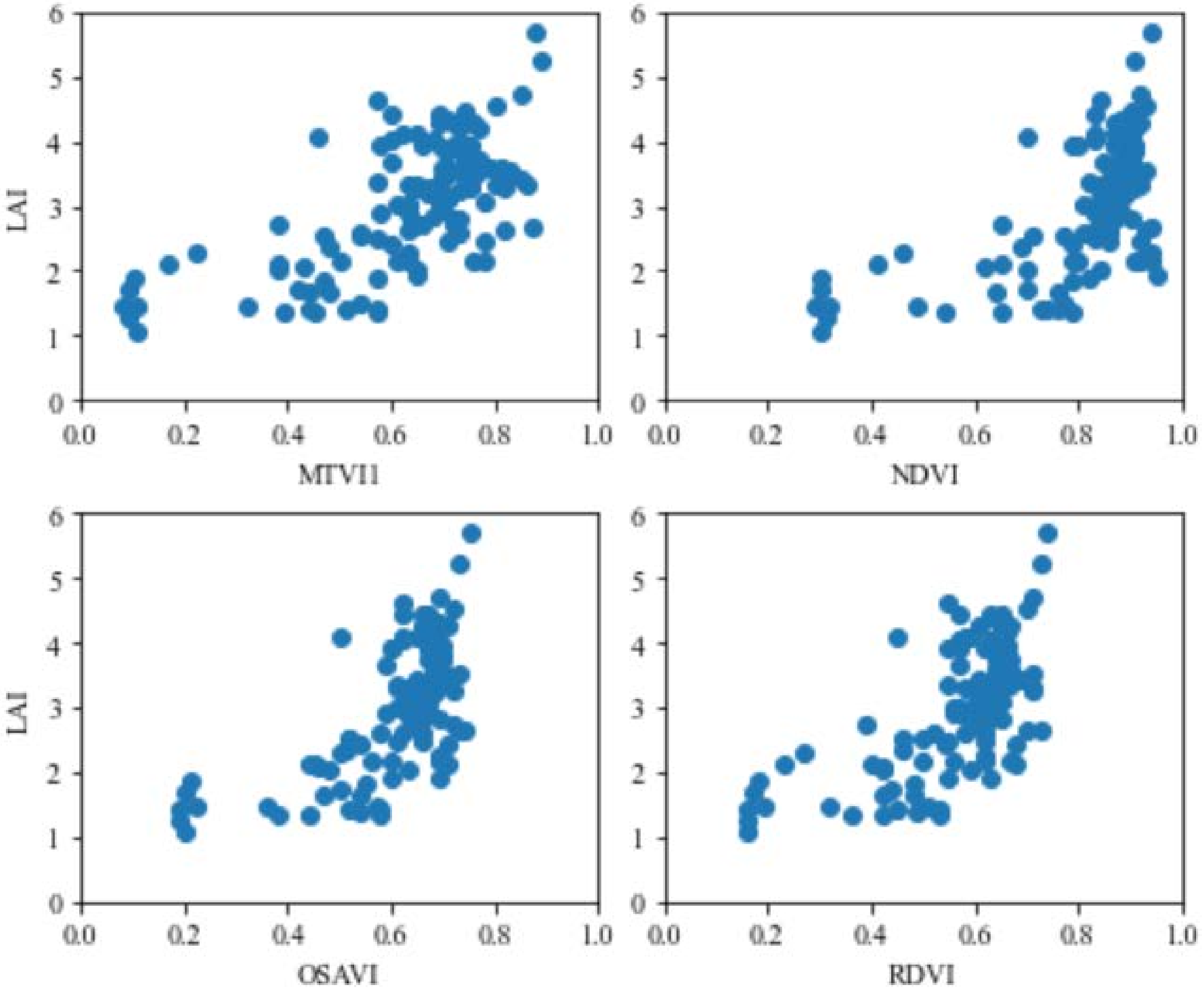

Annex Figure 4. Leaf area index (LAI) versus the four vegetation indices (n = 121) of modified triangular vegetation index 1 (MTVI1), normalized difference vegetation index (NDVI), optimized soil adjusted vegetation index (OSAVI), and re-normalized difference vegetation index.

2 Supplementary Figures

Annex Table 1. Equations applied in remote sensing-integrated wheat model.

Equations	Description
$\Delta D = MAX\ [T-T_b, 0]$	ΔD, daily change in growing degree days (GDD); T, daily mean temperature; T_b, crop specific base temperature
$Q = \beta \cdot R \cdot (1-e^{-k \cdot LAI})$	Q, absorption of incident solar radiation (R); β, fraction of R; k, crop-specific light extinction coefficient; LAI, leaf area index
$\Delta M = \mathcal{E} \cdot Q$	ΔM, daily increase in above-ground dry mass; Ɛ, radiation use efficiency
$\Delta L = \Delta M \cdot P_1 \cdot S$	ΔL, daily LAI increase; P1, fraction of ΔM allocated to new leaves; S, specific leaf area
$P_1 = Max\ [1-a \cdot e^{b \cdot D}, 0]$	P_1, dimensionless leaf allocation function; a and b, parameters controlling magnitude and shape of the function; D, cumulative GDD
$\Delta G = P_2 \cdot \Delta M$	ΔG, daily increase in grain; P_2, fraction of ΔM partitioned to grains
$P_2 = Max\ [1-P_a \cdot e^{P_b \cdot fG_d}, 0]$	P_2, dimensionless grain-partitioning parameter; P_a and P_b, parameters controlling magnitude and shape of the function; fG_d, grain partitioning factor based on cumulative GDD
$\log(VI_t) = \alpha_{VI} + \beta_{VI} \log(LAI_t) + \epsilon_t$	VI_t, vegetation indices; α and β, intercept and slope of the function, respectively; ε, error term
$\Psi = \psi_1, \psi_2, \psi_3, \psi_4 = \left(log\frac{a}{1-a}, log\frac{b}{1-b}, log\frac{c}{1-c}, log\frac{L_O}{1-L_O}\right)$	a and b, parameters in leaf partitioning function; c, parameter in leaf senescence function; L_0, initial LAI

Annex Table 2. Converged parameters of initial leaf area index (L_0) and leaf partitioning and senescence (a, b, and c) after within-season calibration of the remote sensing-integrated wheat model for Chokyung and Keumkang cultivars seeded in the spring and fall of 2018 at Gyeongsang National University (GNU), Jinju, south Gyeongsang province, South Korea, for model calibration.

Season	Cultivar	L_0	a	b	c
Fall	Chokyung	0.8669	0.1699	0.00366	0.00031
Fall	Keumkang	0.9391	0.0121	0.00724	0.00035
Spring	Chokyung	0.6745	0.1804	0.00156	0.04316
Spring	Keumkang	0.7354	0.1211	0.00188	0.06075

Annex Table 3. Converged parameters of initial leaf area index (L_0) and leaf partitioning and senescence (a, b, and c) after within-season calibration of the remote sensing-integrated wheat model for Chokyung wheat seeded in the fall of 2018 at Chonnam National University (CNU), Gwangju, and seeded in the spring and fall of 2019 at Gyeongsang National University, Jinju, south Gyeongsang province, South Korea, with different nitrogen applications of 40 kg ha^{-1} at planting, 30 kg ha^{-1} at rejuvenation, and 0 kg ha^{-1} at initial reproduction (N40-30-0), N40-30-30, and N40-30-60, for model validation.

Season and site	N applied	L_0	a	b	c
Fall, CNU	40-30-30	0.5588	0.6923	0.00032	0.03887
Spring, GNU	40-30-30	1.0000	0.0008	0.00085	0.00933
Fall, GNU	40-30-0	0.9832	0.6784	0.00050	0.02841
	40-30-30	0.7856	0.3826	0.00267	0.00042
	40-30-60	0.7595	0.3737	0.00230	0.00086

Annex Table 4. Descriptive statistical indices (DSI) of mean with standard deviation (SD), maximum, and minimum for two-dimensional variation in simulated values of normalized yield index (NYI), leaf area index (LAI), and above ground dry mass (AGDM) of wheat cultivars grown with different nitrogen (N) gradient treatments in the fall season of 2019 at Gyeongsang National University (GNU), Jinju, South Korea.

N treatment♪	DSI	NYI	LAI	AGDM
		unitless	-- m^2 m^{-2} --	-- g m^{-2} --
N40-0-0	mean ± SD	0.49 ± 0.157	2.6 ± 0.61	781.7 ± 39.79
	max	0.82	4.4	880.7
	min	0.15	1.8	711.1
N40-10-10	mean ± SD	0.41 ± 0.128	2.4 ± 0.47	757.8 ± 27.72
	max	0.73	3.7	843.6
	min	0.13	1.8	706.9
N40-20-20	mean ± SD	0.38 ± 0.142	2.2 ± 0.37	757.6 ± 23.19
	max	0.73	4.4	827.5
	min	0.01	1.8	689.7
N40-30-30	mean ± SD	0.48 ± 0.171	2.3 ± 0.38	766.6 ± 34.13
	max	0.90	4.3	901.4
	min	0.07	1.8	684.5
N40-40-40	mean ± SD	0.56 ± 0.132	2.4 ± 0.33	779.9 ± 30.14
	max	0.86	3.7	877.8
	min	0.14	1.9	707.7
N40-50-50	mean ± SD	0.54 ± 0.143	2.3 ± 0.38	771.9 ± 37.38
	max	0.88	3.8	886.4
	min	0.06	1.7	691.1
N40-60-60	mean ± SD	0.62 ± 0.120	2.5 ± 0.41	791.9 ± 37.55
	max	0.87	3.8	882.3
	min	0.12	1.9	703.7

♪ N40-0-0, N10-10-10, N40-20-20, N40-30-30, N40-40-40, N40-50-50, and N40-60-60 indicate N applications of 40 kg ha^{-1} at planting, 0 to 60 kg ha^{-1} at rejuvenation, and 0 to 60 kg ha^{-1} at initial reproduction.
